# Pancreatic endometrioma : a rare differential diagnosis for a pseudocyst

**DOI:** 10.1259/bjrcr.20220141

**Published:** 2023-01-13

**Authors:** Dana AlNuaimi, Yasir Akmal, Ahmad AlDuaij, Ahmed Sherif, Shareefa Abdulghaffar, Numan Cem Balci

**Affiliations:** 1 Fatima College of Health Sciences, Radiology and Medical Imaging Department, Abu Dhabi, United Arab Emirates; 2 Cleveland Clinic Abu Dhabi, AlMaryah Island, Abu Dhabi, United Arab Emirates; 3 Cleveland Clinic Lerner College of Medicine, Cleveland, USA; 4 Dubai Health Authority, Dubai, United Arab Emirates

## Abstract

Pancreatic endometriosis is extremely rare with only 14 cases reported in the medical literature and its diagnosis on radiological imaging poses a great challenge. We report a case of a 31-year-old female patient with recurrent admissions for pancreatitis of unknown aetiology and no relevant previous medical history. Sectional imaging showed a cystic lesion in the tail of the pancreas and the diagnosis of a post-pancreatitis pseudocyst or a less likely pre-malignant mucinous cystadenoma was considered.

On post-robotic resection of the pancreatic cyst, the histopathology analysis was positive for endometrial stroma. Pancreatic endometriosis although rare should be considered as a differential diagnosis for cystic lesions especially in patients who are known to have pelvic endometriosis. Nevertheless, the gold standard for the definite diagnosis of pancreatic endometriosis remains histopathological.

## Introduction

Endometriosis is defined as the presence of endometrial glandular tissue outside the uterine cavity.^1^ It is a common disease and can be seen in almost 5 to 10% of females in the reproductive age group.^2^ It is also seen in approximately 5% of postmenopausal females especially in patients on estrogen hormonal replacement therapy.^3^ Endometriosis has been linked with a genetic predisposition with findings of estrogen dependence and progesterone resistance.^2^ It is often associated with an inflammatory reaction and resultant fibrotic changes at the site of the endometriotic deposits.^1^

## Case report

A 31 years old female patient presented to the emergency department complaining of epigastric pain associated with nausea and vomiting. She has a past medical history of recurrent episodes of acute pancreatitis and was treated previously in another healthcare facility where no definitive aetiology was found. No other significant past medical history was noted and no cyclical pattern of pain was observed. No previous surgical history was disclosed nor family history of pancreatic diseases or neoplasms was given.

On physical examination, the patients vital signs were normal. Chest was noted to be clear. Abdomen was soft except for mild tenderness in the epigastric region. Laboratory blood work-up showed elevated pancreatic enzymes indicative of pancreatitis. The patient was admitted as a case of acute pancreatitis for further investigation and the treatment was initiated.

**Figure 1.**
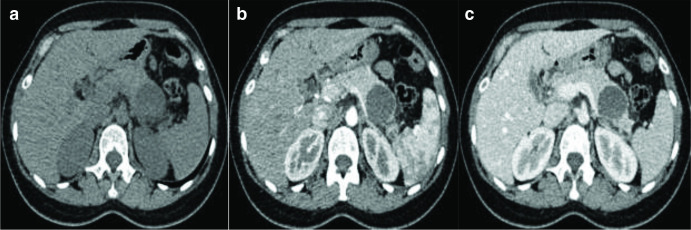
A. Axial non-enhanced CT section of the upper abdomen showing a cystic lesion at the tail of the pancreas. No wall calcifications appreciated. B&C Axial contrast-enhanced CT sections in arterial and porto-venous phases, respectively, showing minimal wall enhancement of the pancreatic cyst with no internal solid component or septations.

A computed tomography (CT) scan of the abdomen and pelvis with IV contrast was done two days after admission and showed no sign of acute pancreatitis, no peripancreatic fatty striation or calcifications. However, a unilocular cystic lesion was seen at the tail of the pancreas measuring approximately 3.6 × 3.3 cm in diameter. Slight atrophy of the distal tail beyond the cyst was notable. No evidence of mesenteric or retroperitoneal lymphadenopathy was seen. Major vasculature appeared intact. (Figure 1)

**Figure 2.**
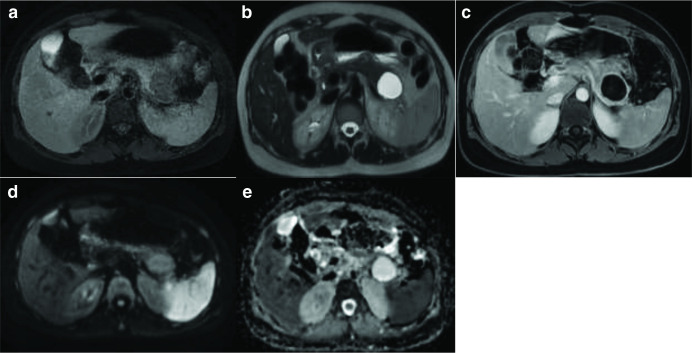
MRI axial sequences of the upper abdomen. A. T1 FATSAT showing a hypointense cystic lesion at the tail of the pancreas. B. T2 HASTE axial sequence showing hyperintensity of the cyst. C.T1 FATSAT post-contrast imaging showing only minimal enhancement of the cyst wall with no internal solid component or septations. D&E. DWI and ADC showing no internal restriction of the cystic lesion.

Gadolinium-enhanced magnetic resonance imaging (MRI) of the abdomen was performed and the results again showed a cystic lesion at the tail of the pancreas which appeared hypointense on T1 FATSAT in comparison with the pancreatic parenchyma and hyperintense on T2 HASTE sequences. There was no restriction of the cystic lesion on diffusion weighted imaging (DWI) and apparent diffusion coefficient (ADC). Minimal contrast enhancement of the wall on post-contrast T1 FATSAT images was depicted. No gradient echo sequences were obtained. (Figure 2)

These findings on diagnostic imaging in addition to the patients history of recurrent pancreatitis suggested a diagnosis of a pseudocyst as a complication of pancreatitis. Nonetheless, a mucinous cystic neoplasm was also included as a differential diagnosis considering the location of the cyst, age, and gender of the patient with the recurrent episodes of pancreatitis possibly due to obstruction to upstream pancreatic duct by the cystic lesion.

The patient underwent an upper esophagogastroduodenoscopy and ultrasound-guided fine needle aspiration of the cystic fluid was done which showed elevated CEA (370 mcg/L) and amylase (621 U l^−1^) levels and was negative for malignant cells. CEA levels above 200 ng ml^−1^ raise suspicion for a mucinous neoplasm, while amylase levels above 200 U l^−1^ raise suspicion of a post-pancreatitis pseudocyst.^4^

The patient underwent a distal subtotal robotic pancreatectomy with splenic preservation. The intraoperative findings showed a cystic 3 cm lesion at the distal pancreatic tail. Splenic artery, splenic vein, and the spleen were preserved.

The histopathology showed a unilocular cyst lined by tall, columnar, non-mucin producing cells with mildly hyperchromatic small nuclei seen focally. Also seen was a multifocal underlying ovarian-type stromal component. In addition to a focally mixed inflammatory infiltrate and foamy histiocytic aggregates with hemosiderin laden macrophages reaching the surface.

**Figure 3.**
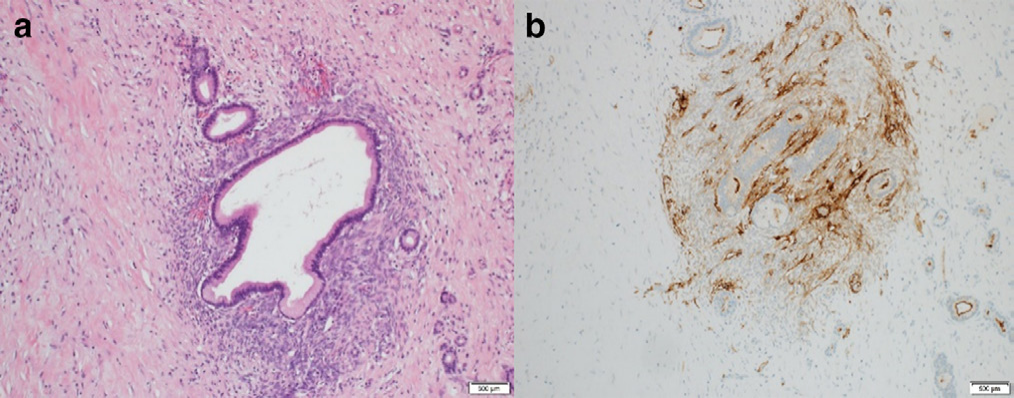
Hematoxylin and Eosin staining of the pancreatic cyst. Original magnification x400. a. Microscopic sectionof subepithelial cystic space show endometrial-type glands with Müllerian type epithelium, partially cyclingendometrium, peri-glandular fibrosis with some atrophy and no significant atypia. b. CD10 immunostain is positivefor endometrial stroma.

Immunohistochemical stains showed spindled cells positive for CD10 and ER and epithelial lining from the cyst expressing CK-7 and CK-19 typical of endometrial stroma. (Figure 3)

The patient had an unremarkable recovery with no complications observed and was discharged with plans for follow-up at the gastroenterology and gynecology outpatient clinics for further evaluation.

## Discussion

Endometriosis can be divided into intrapelvic and extrapelvic disease.^3^ Commonly it is seen in the pelvis near the uterus, fallopian tubes and ovaries and approximately 20 to 25% of the patients are asymptomatic.^5^ Extragenital pelvic manifestations are seen in almost 40% of patients and include rectal deposits, urinary tract, abdominal wall and peritoneal deposits.^2,6^ Extrapelvic endometriosis is only seen in 1% of patients and the diagnosis can be somewhat challenging and is often delayed.^3^ Extrapelvic endometriosis can occur at any location and cases have been described with deposits to the lungs, liver, pancreas, bones and brain.^2^

There are several theories suggesting the pathophysiology of endometriosis and include the direct endometrial tissue extension unto neighboring organs, retrograde menstruation, ectopic production of endometrial stoma from embryonic vestiges, bone marrow and stem cells as well as hematogenous or lymphatic spread.^6,7^

On MRI, ovarian endometriomas have a characteristic homogeneous T1 hyperintensity and a low T2 signal intensity. There may be heterogeneity to the T2 hypointensity and this is referred to as shading and is due to different stages of degradation of the blood products as a result of cyclical episodes of bleeding. Another more specific sign of ovarian endometriomas is called the T2 dark spot sign which appears as discrete markedly hypointense foci in the cyst on *T_2_* weighted images, with or without the T2 shading. In distinguishing an ovarian endometrioma from other non-endometrioma hemorrhagic cystic lesions, a study has shown T2 shading has approximately 93% sensitivity, 45% specificity, 72% positive predictive value (PPV) and 81% negative predictive value (NPV), while T2 dark spots has a 93% sensitivity, 45% specificity, 72% PPV and 81% NPV.^8^

The endometrioma in our case had atypical imaging features with low T1 signal and high T2 signal and therefore was not considered as a differential diagnosis.

Pancreatic endometriosis was first described in the literature in 1984 and is extremely rare with only 14 cases reported up to the current date.^2,6,7,9^ Patients with pancreatic endometriosis usually present with epigastric pain and may be admitted with acute pancreatitis or acute abdomen.^2,7^ The cyclical catamenial nature of symptoms and a past medical history of endometriosis may aid in suggesting the diagnosis.^1,2^ In addition to the frequently changing morphological findings and signal alterations on sectional imaging may also be helpful.^2^

The wide use of sonography, computed tomography, magnetic resonance imaging and positron emission tomography PET resulted in a rise in the incidental recognition of pancreatic lesions in asymptomatic patients.^5,9^ Cystic lesions in the pancreas could be classified into benign, premalignant and malignant cysts.^10^ The differential diagnosis includes post-pancreatitis pseudocysts, mucinous cystic neoplasms, serous cystadenomas or cystadenocarcinomas, cystic pancreatic adenocarcinomas, cystic neuroendocrine tumors and pseudopapillary tumors as well as ectopic tissue such as endometrial cysts.^9,10^

Pancreatic pseudocysts typically form within 6 to 8 weeks after an episode of acute pancreatitis. The imaging features include a round fluid-filled collection and thick enhancing fibrotic wall with no septations, wall calcifications or intracystic solid components. On cystic fluid analysis elevated amylase and lipase levels are commonly encountered with normal CEA levels.^10^

Mucinous cystic neoplasms typically occur in middle-aged female patients and are considered premalignant. Approximately 90% are notable at the pancreatic tail and can reach a size of 20 cm in diameter at the time of diagnosis. They are multilocular and internally-septated with a smooth enhancing contour and wall calcifications. These neoplasms are lined with mucinous epithelium with varying degrees of dysplasia resting on a layer of cellular ovarian-like stroma which mimics endometrial stroma and is usually positive for inhibin, both oestrogen and progesterone receptors but negative for CD 10 on immunochemistry tests which is a common marker for endometrial stroma.^10^

Elevated cyst CEA levels are more common with mucinous cystic neoplasms than non-mucinous lesions and amylase levels are typically normal.^10^

Diagnostic imaging and laboratory tests are valuable tools in the initial assessment of pancreatic endometriosis.^10^ However, imaging is usually non-specific due to overlapping features with premalignant pancreatic lesions especially with the more common mucinous cystic neoplasms.^2,10^ Pancreatic imaging may show cystic lesions of a variable size and complexity with varying degrees of hemorrhagic components.^2^ Magnetic resonance imaging may depict bleeding which appears as hyperintense foci on T1 sequences with/without fat saturation. In the absence of intralesional haemorrhage, hypointense T1 and T2 image signal is usually seen.^2,6^

Up to the current date no typical diagnostic imaging features of pancreatic endometriosis have been established as only a few cases have been reported in the literature.^6^ Nevertheless, hemorrhagic components are extremely uncommon in pancreatic mucinous tumors.^2^ Pancreatic endometriomas may show elevated cyst CEA levels as well as positive CD 10 immunohistochemical marker which is used to denote endometrial stroma.^6,7^ Laparoscopy and histopathological analysis remains the gold-standard for the definitive diagnosis of pancreatic endometriosis.^3^

Ultrasound-guided fine needle aspiration is not recommended if mucinous cystic neoplasms are highly suspected due to the risk of seeding which can cause pseudomyxoma peritonii.^6^ If preoperative suspicion of malignancy cannot be ruled out then distal pancreatic resection with or without splenectomy is recommended.^9^

Definitive diagnosis and treatment of pancreatic endometriosis usually requires a multidisciplinary approach including pancreatic surgeons and gynecologists with the careful surgical excision of all endometrial lesions.^1,9^ Thorough diagnostic investigations including intraoperative frozen section histopathology may avoid extensive surgical resection leading to complications like pancreatic insufficiency.^2,7^ Concomitant evaluation of the pelvic cavity is essential as isolated extrapelvic endometriosis is rare.^1^

In conclusion, pancreatic endometriosis is extremely rare but should be considered as a differential diagnosis for cystic lesions especially in patients who are known to have pelvic endometriosis. Nevertheless, the gold standard for the definite diagnosis of pancreatic endometriosis remains histopathological due to the overlapping radiological imaging features with other pancreatic cystic lesions.

## Learning points

- Endometriosis is a fairly common disease occurring in up to 5–10% of premenopausal females and 5% of postmenopausal females.
- Pancreatic endometriosis is extremely rare and often mimics other pancreatic cystic lesions including pseudocysts, premalignant and malignant cysts.
- Clinical features, radiological imaging and laboratory work-up are essential and may aid in suggesting the diagnosis. However, for a definitive diagnosis surgical resection and histopathology are the gold standard as the radiological image findings of these lesions overlap with other cystic lesions especially premalignant and malignant mucinous neoplasms.

## References

[1] Chamié LP, Ribeiro DMFR, Tiferes DA, Macedo Neto AC de, Serafini PC . Atypical sites of deeply infiltrative endometriosis: clinical characteristics and imaging findings. Radiographics 2018; 38: 309–28. doi: 10.1148/rg.2018170093 29320327

[2] Plodeck V, Sommer U, Baretton GB, Aust DE, Laniado M, Hoffmann R-T, et al . A rare case of pancreatic endometriosis in a postmenopausal woman and review of the literature. Acta Radiol Open 2016; 5: 2058460116669385. doi: 10.1177/2058460116669385 27733937 PMC5040200

[3] Bourgioti C, Preza O, Panourgias E, Chatoupis K, Antoniou A, Nikolaidou ME, et al . MR imaging of endometriosis: spectrum of disease. Diagn Interv Imaging 2017; 98: 751–67: S2211-5684(17)30149-3. doi: 10.1016/j.diii.2017.05.009 28652096

[4] Van Der Waaij LA, Van Dullemen HM, Porte RJ . Cyst fluid analysis in the differential diagnosis of pancreatic cystic lesions: a pooled analysis. Gastrointest Endosc 2005; 62: 383–89. doi: 10.1016/s0016-5107(05)01581-6 16111956

[5] Huang B, Mooser A, Carpenter D, Montenegro G, Luu C . A rare case of pancreatic endometriosis masquerading as pancreatic mucinous neoplasm. Case Rep Surg 2021; 2021: 5570290: 5570290. doi: 10.1155/2021/5570290 34007507 PMC8110415

[6] Yamamoto R, Konagaya K, Iijima H, Kashiwagi H, Hashimoto M, Shindo A, et al . A rare case of pancreatic endometrial cyst and review of the literature. Intern Med 2019; 58: 1097–1101. doi: 10.2169/internalmedicine.1702-18 30568111 PMC6522422

[7] Ludwig C, Kopacz A, Warren ML, Onkendi E . Symptomatic pancreatic body endometrial cyst requiring en bloc distal pancreatectomy. BMJ Case Rep 2021; 14(9): e244911. doi: 10.1136/bcr-2021-244911 PMC8483045 34588203

[8] Corwin MT, Gerscovich EO, Lamba R, Wilson M, McGahan JP . Differentiation of ovarian endometriomas from hemorrhagic cysts at MR imaging: utility of the T2 dark spot sign. Radiology 2014; 271: 126–32. doi: 10.1148/radiol.13131394 24475842

[9] Monrad-Hansen PW, Buanes T, Young VS, Langebrekke A, Qvigstad E . Endometriosis of the pancreas. J Minim Invasive Gynecol 2012; 19: 521–23. doi: 10.1016/j.jmig.2012.03.011 22748958

[10] Mederos MA, Villafañe N, Dhingra S, Farinas C, McElhany A, Fisher WE, et al . Pancreatic endometrial cyst mimics mucinous cystic neoplasm of the pancreas. World J Gastroenterol 2017; 23: 1113–18. doi: 10.3748/wjg.v23.i6.1113 28246486 PMC5311101

